# Recombinant Mammalian Prions: The “Correctly” Misfolded Prion Protein Conformers

**DOI:** 10.3390/v14091940

**Published:** 2022-08-31

**Authors:** Jiyan Ma, Jingjing Zhang, Runchuan Yan

**Affiliations:** Chinese Institute for Brain Research, Beijing 102206, China; zhangjingjing@cibr.ac.cn (J.Z.); yanrunchuan@cibr.ac.cn (R.Y.)

**Keywords:** transmissible spongiform encephalopathies, prion, prion protein, misfolding, recombinant prion, seeding, neurodegeneration, prion disease, prion-like spread

## Abstract

Generating a prion with exogenously produced recombinant prion protein is widely accepted as the ultimate proof of the prion hypothesis. Over the years, a plethora of misfolded recPrP conformers have been generated, but despite their seeding capability, many of them have failed to elicit a fatal neurodegenerative disorder in wild-type animals like a naturally occurring prion. The application of the protein misfolding cyclic amplification technique and the inclusion of non-protein cofactors in the reaction mixture have led to the generation of authentic recombinant prions that fully recapitulate the characteristics of native prions. Together, these studies reveal that recPrP can stably exist in a variety of misfolded conformations and when inoculated into wild-type animals, misfolded recPrP conformers cause a wide range of outcomes, from being completely innocuous to lethal. Since all these recPrP conformers possess seeding capabilities, these results clearly suggest that seeding activity alone is not equivalent to prion activity. Instead, authentic prions are those PrP conformers that are not only heritable (the ability to seed the conversion of normal PrP) but also pathogenic (the ability to cause fatal neurodegeneration). The knowledge gained from the studies of the recombinant prion is important for us to understand the pathogenesis of prion disease and the roles of misfolded proteins in other neurodegenerative disorders.

## 1. Introduction

Transmissible spongiform encephalopathies (TSEs) are a large group of neurodegenerative disorders that includes scrapie in sheep and goats, bovine spongiform encephalopathy in cattle, chronic wasting disease (CWD) in cervids, Creutzfeldt Jakob disease (CJD) and Gerstmann–Sträussler–Scheinker (GSS) disease in humans [1]. Although most TSEs are sporadic, they can also be a genetic or acquired disorder [2]. A unique feature of TSEs is their transmissibility, which separates them from other neurodegenerative disorders and causes epidemic outbreaks, such as the BSE outbreak in Europe and its zoonotic spread that causes a variant of CJD in humans [3] and the ongoing spread of CWD across three continents [4,5].

Since the discovery that TSE is a transmissible disease, the agent responsible for its transmissibility has been extensively studied [6]. The unusual chemical properties of the agent together with the virus-like properties of TSE transmissibility, such as the presence of distinct strains, the ability of a strain to mutate, and the existence of a transmission barrier during inter- or intraspecies transmission, have led to hypotheses that the infectious agent is a slow virus, a subvirus, or a virino [6]. However, these possibilities have been ruled out because there is no nucleic acid specifically associated with TSEs. The term “prion” was coined by Stanley Prusiner for the proteinaceous infectious particles in TSEs with two characteristics: (1) the ability to self-replicate in the absence of a nucleic acid genome and (2) the ability to cause TSEs [7]. The discoveries of the TSE-associated PrP^Sc^ isoform, a misfolded conformer of host-encoded prion protein (PrP) [8,9], and the complete disease resistance of PrP null mice [10] have demonstrated an essential role of PrP in the pathogenic process, but they were insufficient to prove that misfolded PrP^Sc^ is the agent responsible for TSEs. The prion concept has now been expanded to explain a variety of biological phenomena associated with the seeded propagation of various protein conformations [11,12,13]. However, this review focuses exclusively on the originally defined prion—the agent responsible for the transmissibility of TSEs.

The prion hypothesis postulates that the agent is PrP^Sc^, which was originally defined as the proteinase K (PK)-resistant form in diseased individuals [14], but now includes some PK-sensitive misfolded PrP^Sc^ forms [15]. According to the prion hypothesis, PrP^Sc^ coerces host-expressed normal PrP (PrP^C^) to convert to the misfolded PrP^Sc^ isoform, which explains how a prion replicates. Importantly, PrP^Sc^ is also able to initiate the neurotoxic process that ultimately results in neurodegeneration with distinct pathological features. Because it appeared to be incompatible with dogmas in modern molecular biology, this hypothesis was hotly debated for a long time.

**Figure 1:**
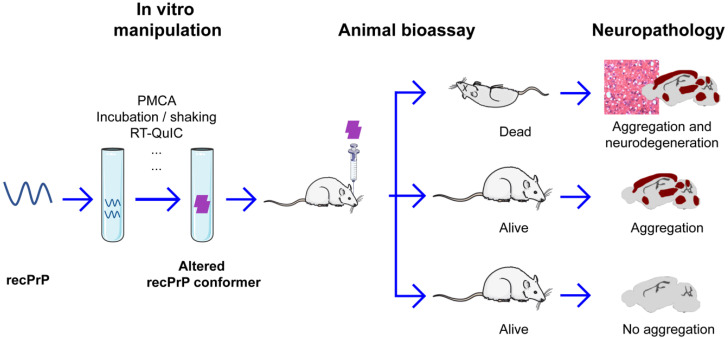
Overview of recombinant prion preparation and animal bioassay.

Koch’s postulates are considered the gold standard to establish the cause-and-effect relationship between an infectious agent and a disease [16]. They require (1) the agent to be isolated from the diseased host and grown in pure culture and (2) the newly generated agent to faithfully reproduce the disease when it is injected into a healthy host. Because PrP^Sc^ is aggregated and surface-exposed hydrophobic amino acids make it “sticky” [7], it cannot be purified to homogeneity either from diseased brains or from in vitro cell cultures. Therefore, the most stringent approach to prove the prion hypothesis needs to fold exogenously produced recombinant PrP (recPrP) to the PrP^Sc^ conformation in a test tube and demonstrate that the in vitro-generated recPrP^Sc^ can seed the PrP^C^-to-PrP^Sc^ conversion and cause bona fide TSEs (also known as prion disease) in a suitable host (Figure 1). In recent years, this approach has led to tremendous successes and provided indisputable evidence to demonstrate that as the prion hypothesis postulated, a misfolded PrP conformer is responsible for the transmissibility of TSEs.

## 2. Exogenously Generated Recombinant PrP

The exogenously prepared recPrP can be produced in bacteria, in insect cells by baculovirus-mediated expression, or in the form of chemically synthesized peptides. Because of its convenience, bacterially expressed recPrP is the most widely used (Table 1). Recombinant PrP is usually expressed in the inclusion body of *E. coli*, which needs to be solubilized, refolded, and chromatographically purified [17]. The refolded recPrP has the same three-dimensional structure and biochemical properties as PrP^C^ [18]. However, it does differ from endogenously expressed PrP^C^ in that it is not glycosylated and without the GPI (glycosylphosphatidylinositol) anchor to tether it to the lipid membranes. The lack of post-translational modifications of recPrP was once considered a great, if not an insurmountable, obstacle to generating a prion in vitro, but a series of studies demonstrated that an authentic prion can be generated with bacterially expressed recPrP [19,20,21,22,23], supporting that the TSE transmissibility is indeed governed by the conformation of PrP. Another difficulty in generating authentic prion in vitro is due to the simple fact that PrP^Sc^ is a misfolded form of PrP. When a protein misfolds, it can misfold into numerous stable conformations and this is also true for PrP. So far, only a small portion of misfolded recPrP conformers appear to possess true prion activity, making it difficult to determine the critical structural features. Here, we review the attempts that generated misfolded conformers with recPrP produced from a non-mammalian source, discuss their implications, and present our views of future directions in this area of study.

## 3. Demonstrating Prion Seeding Activity with recPrP

One of the key properties of a prion is its seeding capability, which was demonstrated by two in vitro assays: (1) the cell-free conversion assay that uses partially purified PrP^Sc^ as the seed and PrP^C^ purified from cultured mammalian cells as the substrate [41] and (2) the protein misfolding cyclic amplification (PMCA) assay that subjects a mixture of diseased and normal brain homogenates to successive sonication/incubation cycles resulting in the propagation of PrP^Sc^ [42]. Both assays demonstrated the capability of PrP^Sc^ to seed PrP^C^-to-PrP^Sc^ conversion, explaining the self-replication property of a prion.

Between these two assays, PMCA is more robust and able to generate a sufficient amount of converted products to cause authentic prion disease in wild-type animals [43]. This protocol was adapted to generate misfolded PrP conformers with bacterially expressed recPrP as the substrate. Atarashi et al. reported the PrP^Sc^-seeded recPrP conversion to a PK-resistant form by PMCA [44] or simply by shaking (named QUIC for “quaking-induced conversion”) [45]. Colby et al. developed an amyloid seeding assay (ASA), which uses partially purified PrP^Sc^ to seed recPrP amyloid fibril growth in a microplate with continuous agitation [46]. Because the appearance of PK-resistant recPrP in the QUIC reaction also correlated with the thioflavin T (ThT) fluorescence signal, the QUIC protocol was further improved to a real-time quaking-induced conversion (RT-QuIC) assay that monitors the seeded conversion of recPrP with ThT fluorescence in real time [47,48]. The RT-QuIC assay has been a great success and has been used extensively in the diagnosis of prion disease [49,50]. More recently, the procedure has been extended to other neurodegenerative disorders [51,52], showing great potential as a sensitive diagnostic assay for a long list of neurodegenerative disorders, including α-synucleinopathies, such as Parkinson’s disease and dementia with Lewy Bodies [53,54,55,56,57,58,59,60], and tauopathies, such as Alzheimer’s disease and primary age-related tauopathy [61,62]. ASA was also adapted to detect the misfolded huntingtin protein in Huntington’s disease [63] and misfolded tau in tauopathies [64], but its application is not as widespread as that of RT-QuIC, possibly due to the cumbersome partial purification steps.

Both RT-QuIC and ASA detect prion-seeding activity based on the seeded growth of recPrP amyloid fibrils, which are highly sensitive and specific. However, they do not fully recapitulate the seeding properties of naturally occurring prions. For example, the recPrP amyloid fibril seedings in these two assays are highly promiscuous and allow efficient cross-species seeding [46,65]. In contrast, naturally occurring prions exhibit strong transmission barriers [66,67,68], and this property was faithfully recapitulated by the classic cell-free conversion and PMCA assays [69,70]. In vitro analyses indicate that the recPrP amyloid growth assay is much more tolerable to amino acid mismatch than PMCA [71].

## 4. In Vitro-Generated recPrP Amyloid Fibrils without Pathogenicity

In addition to seeding, a prion should cause prion disease in a suitable host. Because prion disease is an authentic disease in rodents and prion inoculation in wild-type animals is able to cause fatal neurodegenerative diseases with distinct incubation times, clinical symptoms, and neuropathologies [72], the causal role of in vitro-generated recPrP amyloid fibrils can be clearly determined. A myriad of recPrP amyloid fibrils was generated using different conditions, which resulted in a great variety of outcomes when they were inoculated into the animals. Many recPrP fibrils failed to cause any effect in animals [26,29,38,73,74,75]. Some of them, including seeded recPrP amyloid fibrils generated by RT-QuIC, appeared to be replicated in vivo (inoculated animal brains contained a high number of PrP that were RT-QuIC positive) but still failed to cause clinical disease [29,39].

An interesting example is the study performed by Barron et al. [40]. They used in vitro-prepared recPrP amyloid fibrils to inoculate knock-in mice homozygous for P101L mutant PrP. The human P102L mutation (equivalent to P101L in mouse PrP) caused GSS, which is characterized by the PrP amyloid deposit in the patient’s brain [76]. Inoculations of amyloid fibrils prepared with wild-type or P101L recPrP did not lead to any clinical disease or spongiform changes (classic neuropathological change for prion diseases) but they did cause PrP amyloid deposits in 24/40 animals (Table 1). A subpassage with PrP amyloid-positive brain homogenates resulted in PrP amyloid plagues in 35/47 mice, but again, none of the mice developed any clinical disease or spongiform changes. This study clearly showed that amyloid seeding and deposition in vivo do not necessarily lead to neurodegeneration or clinical disease (Figure 1).

Recent structural studies of protein aggregates in neurodegenerative diseases have revealed that a single protein can form differently packaged amyloid fibrils [77]. It is possible that the particular type of recPrP amyloid fibril used in the above study may not be the type of fibril responsible for the disease. Alternatively, instead of fibrils, the on- or off-amyloidogenic pathway oligomers could be the pathogenic species. In this case, recPrP fibril-seeded PrP amyloid fibril growth may have failed to generate and/or shortened the half-lives of these pathogenic oligomeric species. Nevertheless, although the negative results are generally inconclusive, the above study supports the idea that a protein conformer with seeding ability alone, in vitro or in vivo, does not necessarily mean that it is a prion.

## 5. In Vitro-Generated recPrP Amyloid Fibrils with Atypical Pathogenicity

Transgenic mice over-expressing PrP are more susceptible to prion infection, usually with a shortened incubation time and disease duration [78]. These mice are useful for testing prions with lower infectivity, particularly in vitro-generated recPrP amyloid fibrils or other types of recPrP aggregates. However, the interpretation of the results can be complicated because PrP over-expression not only provides more substrates for PrP conversion but also greatly exacerbates the neurotoxic process in prion disease [79]. In some cases, PrP over-expression alone is sufficient to form a prion de novo [80,81].

Legname et al. showed that inoculating recPrP89-230 amyloid fibrils into mice over-expressing PrP89-231 (at a level that was 16 times that of normal PrP^C^) resulted in neurological disorders in these mice between 380 and 660 days post-inoculation (dpi) [32]. A subpassage of diseased brain homogenate to wild-type FVB mice or mice over-expressing wild-type PrP (at a level 8 times that of normal PrP^C^) led to prion disease with incubation times of 154 and 90 days, respectively. In follow-up studies, multiple types of recPrP amyloid fibrils were generated under varying conditions for amyloid fibril growth. Many of these recPrP amyloid fibrils caused disease in PrP-over-expressing transgenic mice after prolonged incubation periods but none of them directly caused disease in wild-type animals [33,34].

The use of recPrP89-230 is because it is the PK-resistant core of PrP^Sc^, which is sufficient to cause disease in wild-type mice [1]. Full-length recPrP amyloid fibrils typically produce smaller PK-resistant fragments around 10 kDa, which is significantly shorter than that of PrP^Sc^. To extend the PK-resistant fragment, an “annealing” procedure, briefly heating fibrils at 80 °C in the presence of normal brain homogenate or bovine serum albumin, was developed to extend the PK-resistant fragment to around 16 kDa [82]. Makarava et al. reported that although wild-type hamsters inoculated with annealed recPrP fibrils were disease-free during their life span, some animals had PK-resistant PrP^Sc^ in their brains that could be detected by Western blot or PMCA [35]. Further passages in hamsters led to a new SSLOW strain with unique clinical presentation, pathology, and biochemistry. Detailed analyses of recPrP amyloid fibrils, with or without annealing, in passages through wild-type hamsters led these investigators to conclude that in vitro-prepared recPrP amyloid fibrils are significantly different from PrP^Sc^, but they can trigger transmissible prion disease after serial passages in wild-type hamsters by the “deformed templating” mechanism [36,37,83].

Unlike the de novo generation of recPrP amyloid fibrils, the recPrP fibrils formed in the RT-QuIC reaction are seeded by authentic prions [65,84], which presumably recapitulate the structural features of native prion seeds. Groveman et al. systemically analyzed the transmissibility of those fibrils by the intracerebral (i.c.) inoculation of RT-QuIC products into wild-type hamsters or tg7 mice that over-express hamster PrP in a mouse PrP null background [39]. None of the inoculations led to clinical disease, even though some of the animal brains appeared to have aggregated PrP that could seed the RT-QuIC reaction. Interestingly, mutant recPrPs with centrally localized 4 lysines replaced by alanines or asparagines appeared to be more effective in forming PrP aggregates, which is consistent with biochemical analyses showing that these lysine residues play a critical role in modulating PrP misfolding [85,86]. A secondary passage in Tg7 transgenic mice led to clinical disease in some of the mice, possibly through a mechanism similar to “deformed templating” [83].

Other groups also tried many innovative conditions for preparing recPrP amyloid fibrils including conjugating fibrils to magnetic beads to enhance its in vivo persistence, but the i.c. inoculation of these fibrils into wild-type or genetically modified mice failed to cause clinical disease [38,87]. A serial passage of mouse brain homogenates or PMCA products of mouse brain homogenates ultimately led to prion disease and the strain properties of these diseases appeared to be distinct from those caused by the known mouse prion strains [38,87].

Collectively, recPrP amyloid fibrils formed by the classic incubation and/or shaking methods have not been able to cause neurodegenerative disease in wild-type animals. However, they possess seeding abilities, resulting in the propagation of misfolded PrP conformation in a fraction of inoculated animals. These studies suggest that some types of recPrP amyloid fibrils are most likely in a conformational state that is similar but not identical to the conformation of prions. Further adaptation by mechanisms such as “deformed templating” is required to ultimately result in a “correctly” misfolded prion.

## 6. Generating recPrP Conformers with Authentic Seeding Activity and Pathogenicity

The fact that in vitro-formed recPrP amyloid fibrils are unable to act the same as real prions suggests two possibilities: (1) some component is missing and/or (2) the misfolding process by incubation/shaking is not conducive to the formation of an authentic prion. PrP is known to bind a variety of non-protein molecules, including lipids, proteoglycans, and nucleic acids [88,89,90,91], and these interactions may destabilize the α-helical structure of normal PrP^C^ and/or guided PrP misfolding to reach the prion conformation [88,90,92,93]. Compared to amyloid fibril growth using the incubation/shaking method, PMCA is a robust prion propagation reaction that can generate authentic prion infectivity [43]. Wang et al. explored these possibilities and revealed that an authentic prion can be generated by PMCA with bacterially expressed recPrP in the presence of the non-protein cofactors of a synthetic phospholipid POPG (1-palmitoyl-2-oleoylphosphatidylglycerol) and normal mouse liver RNA [19]. In these reactions, recPrP was converted from a soluble and PK-sensitive conformation to an aggregated and PK-resistant state (recPrP^Sc^). Similar to naturally occurring prions, recPrP^Sc^ is able to seed recPrP or PrP^C^ in RT-QuIC and PMCA reactions [19,29], infect susceptible cell lines to establish a chronically infected state [94], and cause bona fide prion disease in wild-type mice via an intracerebral, intraperitoneal, or oral route of infection [19,24,25]. Detailed analyses of recPrP^Sc^-infected mice revealed that the neuroinvasion process and pathological changes, in particular, the highly specific PrP-deposit co-localized ultrastructural membrane changes are consistent with those in authentic rodent prion disease [24]. Moreover, the characteristics of the interspecies transmission of mouse recPrP^Sc^ to a hamster are very similar to that of a known cloned murine prion strain [95] and the infectivity of recPrP^Sc^ can be titrated by both mouse a bioassay and cell culture assay [94]. Structural analyses revealed that recPrP^Sc^ shares structural features with the brain-derived PrP^Sc^ [96]. Thus, recPrP^Sc^ generated through this approach recapitulates all the properties of a naturally occurring prion, not only possessing seeding activity but also causing bona fide prion disease in wild-type animals.

The presence of non-protein cofactors appears to greatly facilitate the conversion from recPrP to the prion conformation. Notably, the total RNA purified from normal mouse liver can be replaced by synthetic polyriboadenylic acid (poly(rA)). Using this approach, Wang et al. showed that the resulting recPrP^Sc^ could infect susceptible cultured cells and cause prion disease in wild-type mice [20], revealing that a prion can be generated with materials entirely from non-mammalian sources, that is, recPrP plus synthetic POPG and poly(rA). Shortly after, Deleault et al. reported another recipe for generating recPrP^Sc^ with non-mammalian materials using recPrP plus synthetic phospholipid PE (phosphatidylethanolamine) through PMCA [21].

The use of non-protein cofactors has led to the question of whether the transmissibility is truly dependent on PrP conformation or those non-protein cofactors. Because the cofactors are required for the misfolding of recPrP, this question is difficult to address but was answered by the discovery that the same PMCA reaction can generate another self-perpetuating PK-resistant recPrP form (named R-low because its PK-resistant fragment is about 1 kDa smaller than that of recPrP^Sc^) [29]. Despite having the same biochemical properties and self-propagating capability as recPrP^Sc^, the R-low form does not cause any clinical disease or neuropathological changes after i.c. inoculation into wild-type mice [29]. Some of the R-low-inoculated mouse brains were RT-QuIC-positive, but another passage in wild-type mice did not cause any clinical disease or neurodegeneration. Because the R-low recPrP form and recPrP^Sc^ were generated with the same PMCA procedure using the same set of recPrP and cofactors [29], the only difference being the recPrP conformation [97], the dramatic difference in the outcomes of the animal bioassay led to the conclusion that prion activity is indeed governed by PrP conformation.

## 7. Converting Insect-Cell-Expressed recPrP to recPrP^Sc^

Unlike bacterially expressed recPrP, insect-cell-expressed recPrP is post-translationally modified by N-linked glycosylation and a GPI anchor [98]. Imamura and colleagues showed that insect-cell-expressed recPrP can be converted to recPrP^Sc^ by PMCA in the presence of protease- and heat-treated insect cell lysates [28]. The recPrP^Sc^ produced by this system not only caused prion disease in wild-type mice but also maintained the strain-specific pathogenic properties of seeds, demonstrating again that an authentic prion can be generated with materials from non-mammalian sources. Using this system, these investigators recently reported that many variants of PK-resistant recPrP could be formed de novo when the temperature was raised to 45 °C and that some of these variants were able to cause prion disease in wild-type mice [99]. Interestingly, prion infectivity appeared to be lost during the serial PMCA propagation, even though the PK-resistant recPrP conformation was stably propagated. This observation suggests that (1) recPrP conformation can be altered (or evolves) during PMCA propagation and (2) similar to the bacterially expressed recPrP discussed above, the seeding activity of misfolded insect-cell-expressed recPrP conformers can be separated from their pathogenicity.

## 8. Generating Vole and Human recPrP^Sc^

Besides commonly used murine or hamster recPrP, bank vole and human recPrP have also been used to create recPrP conformers with both seeding and pathogenic activities [23,31]. The bank vole recPrP^Sc^ was generated by PMCA supplemented with PrP null mouse brain homogenate or individual polyanionic cofactors, and the recPrP^Sc^ generated with this system was sufficient to cause prion disease in wild-type voles [23]. Human recPrP^Sc^ was generated by a plate-formatted quaking-induced conversion reaction with anionic ganglioside GM1 and poly(rA) as cofactors [31]. The converted human recPrP^Sc^ caused prion disease in 6/10 transgenic mice expressing the human PrP N181,197Q mutant that is without N-linked glycosylation but failed to cause disease in transgenic mice expressing wild-type human PrP [31]. This discrepancy could be due to the different efficiencies of human prion strains to induce prion disease in different “humanized” transgenic mouse models [100].

## 9. Generating recPrP^Sc^ without Cofactors

Most studies that have generated recPrP^Sc^ with authentic prion activity have been carried out in the presence of non-protein cofactors. Two reports have shown that recPrP conformers with both transmissibility and pathogenicity could be created in the absence of cofactors. Kim et al. reported that with a modified PMCA procedure, bacterially expressed hamster recPrP could be converted to the PK-resistant form and when inoculated into wild-type hamsters, it caused prion disease in a fraction of the animals (25/47 inoculated hamsters) [27]. Notably, the buffer for the modified PMCA contained anionic detergent sodium dodecyl sulfate (SDS), which is similar to an anionic lipid and can partially replace the function of a lipid cofactor. In another study, Choi et al. studied amyloid fibrils formed by recPrP23-144 [30], which is the same amyloid-forming PrP fragment in GSS patients carrying the Y145stop mutation [76]. The recPrP23-144 spontaneously formed amyloid fibrils under physiological buffer conditions without any cofactors [101] and this type of fibril was sufficient to cause prion disease in wild-type mice [30]. Interestingly, two types of PK-resistant PrP were detected in diseased animal brains [30]—a shorter 6–7 kDa PK-resistant form commonly detected in GSS patients and a longer PK-resistant form typical in prion disease [76]. The simultaneous formation of two different PK-resistant PrP forms raised the possibility that in addition to the propagation of recPrP23-144 amyloid conformation, some type of “deformed templating” occurred to convert full-length PrP^C^ to the PK-resistant PrP^Sc^ form.

Interestingly, using full-length recPrP and phospholipid PE as cofactors, Deleault et al. showed that removing PE from the recPrP^Sc^ propagation reaction resulted in a protein-only PK-resistant recPrP with a PK-resistant core similar to that of the R-low form. This recPrP form could be propagated indefinitely by PMCA but failed to cause any disease when i.c. inoculated into animals [26]. The stark differences between these studies could be due to the variabilities in preparing the misfolded recPrP conformers, such as the variability in the type of recPrP, recPrP refolding and purification method, and substrate preparation, and whether the preparation was with or without SDS, as well as the power of sonication, etc.

## 10. De Novo Versus Seeded Formation of recPrP^Sc^

In several studies, recPrP^Sc^ appears to be formed de novo in unseeded reactions [19,22,23,99]. In two independent attempts, Wang et al. and Zhang et al. performed PMCA with recPrP plus POPG and mouse liver RNA in two labs and both generated recPrP^Sc^ de novo [19,22]. A detailed comparison of these independently produced recPrP^Sc^ revealed clear differences in their biochemical and pathological properties [102], supporting the idea that these two recPrP^Sc^ were formed independently. Besides murine recPrP, Fernandez-Borges et al. showed that vole recPrP supported de novo recPrP^Sc^ formation [23].

Interestingly, a self-perpetuating, PK-resistant recPrP conformer with a 14 kDa PK-resistant fragment was also generated de novo in a serial PMCA reaction [22]. Similar to the R-low recPrP form, the 14 kDa PK-resistant recPrP conformer failed to cause any clinical disease in wild-type mice. Interestingly, a similar nonpathogenic PrP conformer with a 14 kDa PK-resistant fragment was isolated from diseased sheep [103], suggesting that this nonpathogenic, self-perpetuating PrP conformer might be one of the preferred misfolded PrP conformations and is present in individuals suffering from natural prion disease.

The efficiency of forming recPrP^Sc^ de novo appears to be low, which is probably consistent with the low incidence of sporadic prion disease [104] and suggests that de novo prion formation by PMCA is a stochastic process. In addition, a PMCA reaction is highly variable and can be influenced by numerous factors, including recPrP refolding and purification, the components of the substrate mixture, temperature, sonication power, length of sonication, number of cycles, wear and tear of the sonicator, etc. Because of the powerful seeding activity of a prion, an optimized PMCA reaction is consistent in detecting prion seeding activity. However, for de novo prion formation, all these variables may affect the process, making the stochastic process even more difficult to “correctly” misfold recPrP. Notably, a shaking procedure was developed to propagate recPrP^Sc^, which eliminates many variables associated with sonication [105]. Theoretically, it would be a more consistent approach to study de novo prion formation, but it remains unclear whether the new shaking method is able to form recPrP^Sc^ de novo.

In contrast to de novo prion formation, generating recPrP^Sc^ with a native prion seed is supposed to recapitulate the structural features of the native prion seed. Although the faithful seeding of native prions has been reported [28], it does not have a clear advantage in producing authentic recPrP^Sc^ (Table 1). Several reasons may account for this discrepancy. First, the in vitro recPrP conversion system is different from the in vivo environment and many factors, including salinity, temperature, and pH, can influence the conformation of recPrP. As a result, the final misfolded recPrP could be different from the native prion seed. Second, non-protein cofactors are known to greatly influence PrP conversion [106], but the identity of the cofactor(s) for each type of prion remains unclear. The current recPrP conversion system uses several commonly used cofactors, which could be different from the real in vivo situation in the types and/or quantities of the cofactors. This difference could also explain why the PMCA propagation of native prion seeds with cofactors supplied by PrP null mouse brain homogenate appears to be quite efficient [23,98].

## 11. The Potential Role of Non-Protein Cofactors in Generating recPrP^Sc^

To date, all de novo recPrP^Sc^ formation requires the presence of non-protein cofactors, and aside from the one study discussed above [27], all propagation of recPrP^Sc^ with authentic prion activity also requires the presence of cofactors. However, the molecular mechanism underlying the cofactors’ effect on recPrP^Sc^ formation remains unclear [106]. Unlike other aggregated proteins such as α-synuclein and tau that are naturally unfolded, the C-terminal part of recPrP is well folded [107]. Therefore, the first step in the PrP^C^-to-PrP^Sc^ conversion requires the removal or destabilization of the normal α-helical structure of PrP^C^. It has been shown that the binding of cofactors such as anionic phospholipid POPG caused a substantial change in the recPrP conformation and destabilized the α-helical structure of recPrP [88,93,108], which potentially allowed it to reach various misfolded forms. Non-protein cofactors could also contribute to the process that the unfolded recPrP acquires the misfolded conformation. The observation that adding another cofactor RNA to the recPrP-POPG complex led to further structural arrangements and the exposure of the N-terminus of recPrP [93] is consistent with this idea.

The requirement of the cofactor in forming a prion could offer a plausible explanation for the peculiar prion strain phenomenon. Prion strains are classified based on the clinical manifestations, pathologies, and biochemical properties of PrP^Sc^ [72,109]. PrP molecules with identical amino acid sequences were postulated to misfold into PrP^Sc^ conformers with minor but distinct structural differences, resulting in different prion strains [109,110,111]. Since a variety of cofactor molecules have been identified, different cofactors may guide PrP into different misfolding processes and/or stabilize different final PrP^Sc^ structures. Consistent with this hypothesis, when a single cofactor PE was used to propagate recPrP^Sc^ seeded by three prion strains, the strain properties converged to a single prion strain [26]. When PrP null mouse brain homogenate in the recPrP^Sc^ propagation reaction was replaced by different polyanionic cofactors, the recPrP^Sc^ diverged into different conformers with distinct strain properties [23].

Together, the non-protein cofactors appear to play three roles in recPrP misfolding: (1) destabilizing the recPrP structure, (2) guiding the recPrP misfolding process, and (3) stabilizing the final recPrP^Sc^ structure that governs the specific disease phenotypes or prion strain properties.

## 12. Summary and Perspectives

All major studies that generated misfolded recPrP forms are summarized in Table 1 and clearly show that all misfolded recPrP forms have in vitro seeding capabilities and many of them have in vivo seeding activities as well; however, seeding ability alone is not sufficient to cause fatal neurodegeneration in wild-type animals (Table 1 and Figure 1). The results of these studies also indicated that the inclusion of non-protein cofactor(s) and the use of the PMCA approach correlate well with the generation of recPrP^Sc^ (Table 1), supporting the critical role of non-protein cofactors and proper in vitro manipulation. The finding that a great number of recPrP amyloid fibrils failed to cause prion disease in wild-type animals is probably consistent with the fact that the majority of sporadic prion diseases do not have PrP amyloid fibril deposition [112,113], which reinforces the idea that only a small number of “correctly” misfolded PrP conformers are the true culprit for the disorder. Nevertheless, the generation of recPrP^Sc^ with authentic prion activity provided unequivocal evidence to prove that the transmissible agent in prion disease is a misfolded PrP conformer, which is sufficient to seed the misfolding of endogenous PrP^C^ and initiate the neurotoxic process leading to a fatal neurodegenerative disease.

Despite great advances, many important questions remain to be answered in the prion field and the simplicity of the recPrP conversion system may help to address some of these questions. One of the fundamental questions is the structural basis for prion infectivity, which could potentially help us understand the peculiar properties of prion transmission, including the strains, prion mutations, and transmission barriers. Several structures of recPrP amyloid fibrils and PrP fibrils isolated from diseased brains were reported recently [114,115,116,117,118,119]. Even though these are great steps toward the ultimate goal, we are still far away from thoroughly understanding the structural basis of prion transmissibility. Moreover, as discussed above, a true prion conformer may not be the major species in the PrP aggregate and this could be a challenge in identifying the critical structural features that are relevant to prion transmissibility. Second, the convenience of in vitro recPrP^Sc^ propagation assays allows us to dissect the primary amino acid sequence of PrP to determine its influence on the susceptibility and resistance to form a particular prion strain. Some studies have explored this possibility [120,121,122,123], but a coherent picture is still lacking. Third, studies of recPrP^Sc^ in vitro demonstrated the importance of non-protein cofactors in forming and maintaining a particular recPrP^Sc^ structure, but the identity of the real cofactors in vivo remains unknown. At the same time, it is also unclear whether different sets of cofactors are responsible for different prion strains, whether prions from different animal species use the same or different cofactors, and whether there is a switch of cofactor(s) during prion mutation. The in vitro recPrP^Sc^ propagation assay provided a great tool to test and characterize the candidates for the in vivo cofactors. Finally, prion disease is still an incurable fatal disease and the in vitro recPrP^Sc^ system could help the development of effective therapeutic or preventive measures against these disorders.

Besides prion disease, the prion concept has been extended to other neurodegenerative diseases characterized by the deposition of misfolded proteins [124,125]. The prion-like spread of ordered protein aggregates has been demonstrated with a variety of misfolded proteins in animal models and humans, and some of these studies have created excellent disease models, such as the alpha-synuclein preformed fibril model for alpha-synucleinopathies [126]. However, similar to the discussion here and one in a recently published review [127], the association between the seeded propagation of misfolded proteins and the real pathogenic process needs to be carefully evaluated. Further studies in this area may ultimately allow us to untangle the intricate relationship between misfolded proteins and neurodegenerative disorders.

## Figures and Tables

**Table 1:** Studies of misfolded recPrP conformers.

Source of recPrP	In Vitro Manipulation	Cofactor	Products	Seeding Activity			Recipient Animal	Route of 1st Passage	Neurodegeneration			Refs.
				In Vitro		In Vivo			Incubation (i) or Survival (s) Time for 1st Passage (Attack Rate)	Incubation (i) or Survival (s) Time for 2nd Passage by i.c. Route (Attack Rate)	Incubation (i) or Survival (s) Time for 3rd Passage by i.c. Route (Attack Rate)	
				with recPrP	with Native PrP							
mouse recPrP from *E. coli*	unseeded serial PMCA	POPG + mouse liver RNA	recPrP^Sc^	Yes (PMCA with recPrP; RT-QuIC)	Yes (PMCA with mouse brain homogenate; infecting cultured cells)	Yes	wild-type mice	i.c.	150 ± 2.2 days (s) (100%)	166 ± 1.5 days (s) (100%)	⎯⎯⎯	[19]
								i.p.	206.8 ± 3.8 days (s) to 220 ± 1.79 days (s) (100%)	156.3 ± 2.3 days (s) to 184.8 ± 13.2 days (s) (100%)	⎯⎯⎯	[24]
				Yes (PMCA with recPrP)	⎯⎯⎯			Oral	194 days (s) (1/11)	191 ± 5 days (s) (100%)	⎯⎯⎯	[25]
mouse recPrP from *E. coli*	seeded serial PMCA	POPG + poly(rA)	recPrP^Sc^	Yes (PMCA with recPrP)	Yes (Infecting cultured cells)	Yes	wild-type mice	i.c.	220 ± 4.5 days (s) and 228 ± 4.5 days (s) (100%)	172 ± 5.4 days (s) and 173 ± 2.6 days (s) (100%)	⎯⎯⎯	[20]
mouse recPrP from *E. coli*	seeded serial PMCA	plasmalogen PE	recPrP^Sc^	Yes (PMCA with recPrP)	⎯⎯⎯	Yes	wild-type mice	i.c.	381 ± 11 days (i) (100%)	175 ± 4 days (i) (100%)	⎯⎯⎯	[21]
mouse recPrP from *E. coli*	seeded serial PMCA	purified mouse brain phospholipids	recPrP^Sc^	Yes (PMCA with recPrP)	Yes (PMCA with mouse brain homogenate)	Yes	wild-type mice	i.c.	356 ± 12 days (i) (100%)	175 ± 4 days (i) (100%)	⎯⎯⎯	[26]
		⎯⎯⎯	Protein-only recPrP-res	Yes (PMCA with recPrP)	No	No	wild-type mice	i.c.	No disease	⎯⎯⎯	⎯⎯⎯	
mouse recPrP from *E. coli*	unseeded serial PMCA	POPG + mouse liver RNA	recPrP^Sc^	Yes (PMCA with recPrP)	⎯⎯⎯	Yes	wild-type mice	i.c.	172.3 ± 1.6 days (s) (100%)	161.3 ± 1.8 days (s) (100%)	⎯⎯⎯	[22]
			14 kDa recPrP-res	Yes (PMCA with recPrP)	⎯⎯⎯	No	wild-type mice	i.c.	No disease	⎯⎯⎯	⎯⎯⎯	
hamster recPrP (90-231 or full-length) from *E. coli*	seeded serial PMCA	⎯⎯⎯	recPrP^Sc^	Yes (PMCA with recPrP)	⎯⎯⎯	Yes	wild-type hamsters	i.c.	162 ± 16 days (i) to 328 ± 113 days (i) (25/47)	75 ± 4 days (i) to 84 ± 1 days (i) (100%)	⎯⎯⎯	[27]
mouse recPrP from insect cells	seeded serial PMCA	PK- and heat-treated insect cell lysates	recPrP^Sc^	Yes (PMCA with insect-cell-expressed recPrP)	⎯⎯⎯	Yes	wild-type mice	i.c.	162 ± 9 days (i) for Chandler-seeded and 193 ± 11 days (i) for mBSE-seeded (100%)	⎯⎯⎯	⎯⎯⎯	[28]
mouse recPrP from *E. coli*	seeded serial PMCA	POPG + mouse liver RNA	recPrP^Sc^	Yes (PMCA with recPrP; RT-QuIC)	Yes (Infecting cultured cells)	Yes	wild-type mice	i.c.	172.2 ± 1.1 days (s) (100%)	⎯⎯⎯	⎯⎯⎯	[29]
			R-lowrecPrP-res		No	Yes (by RT-QuIC)	wild-type mice	i.c.	No disease	No disease	⎯⎯⎯	
vole recPrP from *E. coli*	seeded and unseeded serial PMCA	PrP null mouse brain homogenate	recPrP^Sc^	Yes (PMCA with recPrP)	Yes (PMCA of vole or tgVole mouse brain homogenate)	Yes	wild-type bank voles with I109	i.c.	133 ± 5 days (s) to 172 ± 6 days (s) (63–100%)	61 ± 1 days (s) to 103 ± 4 days (s) (100%)	⎯⎯⎯	[23]
	seeded serial PMCA	dextran, RNA, plasmid DNA, or no cofactor	recPrP^Sc^	Yes (PMCA with recPrP)	Yes (PMCA of vole or tgVole mouse brain homogenate)	Yes	wild-type bank voles with I109	i.c.	157 ± 6 days (s) to 424 ± 51 days (s) (78–100%)			
mouse recPrP23-144 from *E. coli*	incubation at 25 °C	⎯⎯⎯	recPrP23-144 amyloid fibrils	Yes (recPrP amyloid fibril growth)	Yes (PMCA of mouse brain homogenate)	Yes	wild-type mice	i.c.	543 ± 54 days (i) (100%)	⎯⎯⎯	⎯⎯⎯	[30]
							tga20 mice (8X level of PrP)	i.c.	254 ± 12 days (i) (100%)	215 ± 19 days (i) (100%)	208 ± 10 days (i) (100%)	
Human recPrP from *E. coli*	seeded quaking-induced conversion	GM1 + poly(rA)	rhuPrP^Sc^	Yes (QuIC)	⎯⎯⎯	Yes	TgNN6h mice (0.6X level of PrP)	i.c.	459 ± 114 days (i) (6/10)	224 ± 6 days (i) (100%)	⎯⎯⎯	[31]
							Tg40 mice (1X level of PrP)	i.c.	No disease	⎯⎯⎯	⎯⎯⎯	
mouse recPrP89-230 from *E. coli*	incubation at 37 °C with shaking	⎯⎯⎯	recPrPamyloid fibrils	Yes (recPrP amyloid fibril growth)	⎯⎯⎯	Yes	Tg9949 mice (Expressing PrP89-231 at 16X level of PrP)	i.c.	516 ± 27 days (i) and 590 ± 46 days (i) (100%)	258 ± 25 days (i) (100% in Tg9949 mice)	⎯⎯⎯	[32]
										154 ± 4 days (i) (100% in wild-type mice)		
										90 ± 1 days (i) (100% in Tg4053 mice expressing 8X PrP)		
mouse recPrP89-230 and recPrP23-230 from *E. coli*	incubation under various conditions	⎯⎯⎯	recPrP amyloid fibrils	Yes (recPrP amyloid fibril growth)	⎯⎯⎯	Yes	Tg4053 mice (Expressing 8X PrP)	i.c.	554 ± 14 days (i) to 689 ± 33 days (i) (10 of 11 types of recPrP fibrils caused disease or appearance of PrP^Sc^ in the brain detected by WB or ASA)	110 ± 5 days (i) to665 ± 10 days (i) (100% in Tg4053 mice expressing 8X PrP)	⎯⎯⎯	[33]
										144 ± 4 days (i) to585 ± 13 days (i) (In wild-type mice, 4/6 types caused disease with 100%; 2/6 types did not cause disease)		
mouse recPrP89-230 from *E. coli*	incubation under various conditions	⎯⎯⎯	recPrPamyloid fibrils	Yes (recPrP amyloid fibril growth)	⎯⎯⎯	Yes (by ASA)	Tg9949 mice (Expressing PrP89-231 at 16X level of PrP)	i.c.	496 to 669 days (s) (23/26 types of fibrils caused disease with attack rates from 67–100%. 3/26 failed to cause disease	559 ± 12 days (i) to 598 ± 13 days (i) (100%)	⎯⎯⎯	[34]
						No	wild-type mice		No disease	⎯⎯⎯		
hamster recPrP from *E. coli*	incubation at 37 °C with shaking	Annealed with normal brain homogenate or BSA	recPrPamyloid fibrils	Yes (recPrP amyloid fibril growth)	⎯⎯⎯	Yes	wild-type hamsters	i.c.	No disease (1/7 had atypical PrP-res detected by WB; 3/7 had PrP-res detected by serial PMCA)	481 ± 4 days (i) (100% by brain homogenate prepared from the mouse with PrP^Sc^ detected by WB) 565 ± 14 days (i) (100% by brain homogenate prepared from the mouse with PrP^Sc^ detected by PMCA)	⎯⎯⎯	[35]
hamster recPrP from *E. coli*	incubation at 37 °C with shaking	Annealed with BSA	recPrPamyloid fibrils	Yes (recPrP amyloid fibril growth)	⎯⎯⎯	Yes	wild-type hamsters	i.c.	No disease (1/7 had atypical PrP-res detected by WB; 3/7 had PrP-res detected by serial PMCA)	No disease (6/7 had a mixture of typical and atypical PrP-res detected by WB; all 7 had typical PrP-res detected by PMCA)	~10-12 months (i) (12/12)	[36]
hamster recPrP from *E. coli*	incubation at 37 °C with shaking or rotating	⎯⎯⎯	recPrPamyloid fibrils	Yes (recPrP amyloid fibril growth)	⎯⎯	Yes	wild-type hamsters	i.c.	No disease (Some animals had a mixture of typical and atypical PrP-res detected by WB)	347 ± 7 days (i) to512 ± 82 days (i) (71% -100%)	⎯⎯⎯	[37]
mouse recPrP from *E. coli*	incubation under various conditions	⎯⎯⎯	recPrPamyloid fibrils	Yes (recPrP amyloid fibril growth)	Yes (PMCA with mouse brain homogenate; infecting cultured cells)	Yes (by PMCA)	wild-type mice	i.c.	No disease (Only mice that received one type of fibrils had seeding activity for serial PMCA)	No disease (Positive PMCA products from 1st passage caused disease in 130 ± 4 days (i))	⎯⎯⎯	[38]
hamster recPrP from *E. coli*	seeded RT-QuIC	⎯⎯⎯	recPrPamyloid fibrils	Yes (RT-QuIC)	⎯⎯⎯	Yes	wild-type hamsters	i.c.	No disease (12/12 RT-QuIC-positive)	⎯⎯⎯	⎯⎯⎯	[39]
							tg7 mice (Over-expressing hamster PrP in mouse PrP null background)	i.c.	No disease (12/12 RT-QuIC-positive; one mouse showed atypical PrP-res detected by WB)	143 -251 days (s) (5/5)	⎯⎯⎯	
hamster recPrP K4 mutants from *E. coli*							wild-type hamsters	i.c.	No disease (17/17 RT-QuIC-positive; one animal showed atypical PrP-res detected by WB)	⎯⎯⎯	⎯⎯⎯	
							tg7 mice (Over-expressing hamster PrP in mouse PrP null background)	i.c.	No disease (4/6 RT-QuIC-positive; 4/5 showed atypical PrP-res detected by WB)	101–433 days (s) (9/14 clinical signs; 14/14 RT-QuIC-positive; 14/14 had typical PrP-res by WB)	⎯⎯⎯	
mouse recPrP from *E. coli*	incubation with shaking	⎯⎯⎯	recPrPamyloid fibrils	Yes (recPrP amyloid fibril growth)	⎯⎯⎯	No	wild-type mice	i.c.	No disease	⎯⎯⎯	⎯⎯⎯	[40]
						Yes	101LL knock-in mice	i.c.	No disease (10/21 had amyloid deposit)	No disease (17/23 had amyloid deposit)	⎯⎯⎯	
recPrP P101L mutant from *E. coli*						No	wild-type mice	i.c.	No disease	⎯⎯⎯	⎯⎯⎯	
						Yes	101LL knock-in mice	i.c.	No disease (14/19 had amyloid deposit)	No disease (18/24 had amyloid deposit)	⎯⎯⎯	

PrP-res, PK-resistant PrP form; recPrP-res, PK-resistant recPrP form; rhuPrP^Sc^, scrapie form of recombinant human PrP; PMCA, protein misfolding cyclic amplification; RT-QuIC, real-time quaking-induced conversion; QuIC, quaking-induced conversion; ASA, amyloid seeding assay; WB, western blot; 101LL knock-in mice, homozygous knock-in mice expressing P101L mutant PrP. 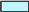 Preparing misfolded recPrP conformers with PMCA. 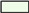 Preparing misfolded recPrP conformers by incubation/shaking. 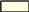 Preparing misfolded recPrP with non-protein cofactors or “annealing” recPrP amyloid fibrils with brain homogenates or BSA. 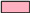 Recombinant PrP conformers that caused disease in the first passage in wild-type animals. 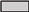 Misfolded recPrP conformers with in vitro seeding activity. 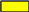
Misfolded recPrP conformers with in vivo seeding activity.
